# Anti-Biofilm Activity of Cell Free Supernatants of Selected Lactic Acid Bacteria against *Listeria monocytogenes* Isolated from Avocado and Cucumber Fruits, and from an Avocado Processing Plant

**DOI:** 10.3390/foods11182872

**Published:** 2022-09-16

**Authors:** Reabetswe D. Masebe, Mapitsi S. Thantsha

**Affiliations:** Department of Biochemistry, Genetics and Microbiology, University of Pretoria, Pretoria 0002, South Africa

**Keywords:** *Listeria monocytogenes*, biofilm, cell free supernatants, avocado, cucumber

## Abstract

*Listeria monocytogenes* forms biofilms on food contact surfaces, a niche from where it dislodges to contaminate food products including fresh produce. Probiotics and their derivatives are considered promising alternative strategies to curb the presence of *L. monocytogenes* in varied food applications. Nonetheless, studies on their anti-biofilm effects against *L. monocytogenes* from avocados and cucumbers are sparse. This study screened the biofilm formation capabilities of *L. monocytogenes* strains Avo and Cuc isolated from the avocado and cucumber fruits respectively, and strain 243 isolated from an avocado processing plant; and evaluated the anti-biofilm effects of cell free supernatants (CFS) of *Lactobacillus acidophilus* La14 150B, *Lactiplantibacillus plantarum* B411 and *Lacticaseibacillus rhamnosus* ATCC 53103 against their biofilms formed on polyvinyl chloride (PVC) and stainless steel. All the *L. monocytogenes* strains formed biofilms (classified either as moderate or strong biofilm formers) on these materials. The presence of CFS reduced the biofilm formation capabilities of these strains and disrupted the integrity of their pre-formed biofilms. Quantitative reverse transcriptase polymerase chain reaction revealed significant reduction of positive regulatory factor A (*prfA)* gene expression by *L. monocytogenes* biofilm cells in the presence of CFS (*p* < 0.05). Thus, these CFS have potential as food grade sanitizers for control of *L*. *monocytogenes* biofilms in the avocado and cucumber processing facilities.

## 1. Introduction

*Listeria monocytogenes* is a Gram positive, foodborne pathogen that has been linked to incidents of severe foodborne illnesses from isolated infections as well as from those connected to foodborne disease outbreaks [1]. *L. monocytogenes* poses a serious concern due to its adaptability features and prevalent nature in many stress conditions and different food storage areas. It is transmitted to humans through consumption of contaminated food such as meat, poultry, dairy (e.g., unpasteurized milks, cheeses, ice cream), ready-to-eat (RTE) foods (e.g., hot dogs, deli meats and smoked fish), fruits and vegetables [2,3].

*L. monocytogenes* has the incredible ability to form biofilms, which are three-dimensional architectural structures made up of a matrix composed of extracellular polymeric substances (EPS), phospholipids, proteins and extracellular DNA [4,5]. The formation of biofilms by *L. monocytogenes* [6], together with the expression of most of the known listerial virulence genes necessary for its persistence and intracellular dissemination [7] are regulated and controlled by the Positive Regulatory Factor A (PrfA) protein encoded by the *prfA* gene. Biofilms are categorized as the most widespread mode of growth in both natural and industrial realms and provide protection to harsh environments [8], as well as facilitate bacterial growth and survival [4].

The presence of *L. monocytogenes* biofilms on food-contact surfaces present a consistent source of contamination and thus a food safety risk [7]). The biofilm formed on these hydrophilic and hydrophobic surfaces promotes persistence of *L. monocytogenes* in different environments, including food-processing environments [9]. *L. monocytogenes* biofilms adhere to various surfaces including polytetrafluoroethylene (PTFE) used in conveyor belts; polyester used as a floor sealer; stainless steel used for the majority of the equipment, polystyrene as a material for the drains; rubber used in joints; wood and also glass [10,11]. *L. monocytogenes* is most often transferred from these surfaces into foods, to ultimately be ingested by consumers. Subsequent to its ingestion, it causes a disease called listeriosis, which is particularly severe in pregnant, the elderly, young (particularly neonates) and the immunocompromised individuals [7,12]. Its mortality rate is the highest amongst all other foodborne pathogens. The worst ever global outbreak of listeriosis was reported in South Africa in the years 2017–2018, with more than 1000 cases reported, of which 200 were fatal [13].

The complex structure of biofilms offers the microbes enclosed within an adaptive and resistance strategy, which protects them from antimicrobial compounds [4,14,15]. This development of antimicrobial resistance has necessitated the exploration of alternative strategies for biofilm control, including among others, the use of essential oils, active packaging, bio-protection and probiotics and their derivatives [15,16]. Probiotics and their derivatives are evidently the strongest and most promising alternative strategy for control of bacterial biofilms [16]. They have also been shown to possess the anti-listerial activity [17]. Despite studies reporting the transmission of *L*. *monocytogenes* through fruits and vegetables [18,19,20]), specifically avocados [21] and cucumbers [22]; and the possession of anti-listerial properties by probiotics [4,14,16], there are no studies reporting the efficacy of probiotics in managing *L. monocytogenes* biofilms in avocado and cucumber processing plants. In light of these, this study aimed to screen the biofilm formation capabilities of *L. monocytogenes* strains from the avocado and cucumber environments and to evaluate the anti-biofilm effects of cell free supernatants (CFS) of selected lactic acid bacteria against the biofilms formed by these strains on different simulated food contact surfaces. Furthermore, this study compared the expression of the positive regulatory factor A (*prfA*) gene by *L. monocytogenes* biofilm cells in the presence and absence of the CFS of the test LAB strains.

## 2. Materials and Methods

### 2.1. Bacterial Strains, Culture Media and Growth Conditions

*Listeria monocytogenes* Avo and Cuc strains previously isolated from avocado and cucumber, respectively, and *L*. *monocytogenes* 243 isolated from an avocado processing plant [23] and *L. monocytogenes* ATCC 19115 were used. Since the study aimed to also fill the existing gap in knowledge with regards to the potential for use of probiotics for control of *L. monocytogenes* associated with avocados and cucumbers, strains Avo, Cuc and 243 were selected due their association with these fresh produce, while ATCC 19115 was used as a positive control. All these strains were grown on Listeria-enrichment agar plates and then sub-cultured twice into Brain Heart Infusion (BHI) broth and incubated at 37 °C for 24 h before their use in experiments. The glycerol stocks of two commercial probiotic strains, *Lactobacillus acidophilus* La14 150B (Danisco Inc., New Century, KS, USA) and *Lacticaseibacillus rhamnosus* ATCC 53103 (American Type Culture Collection, Manassas, Virginia, USA) as well as a potential probiotic strain *Lactiplantibacillus plantarum* B411 isolated from a fermented cereal (Council for Scientific and Industrial Research, Pretoria, Gauteng, South Africa), were used as lactic acid bacteria (LAB) test cultures. They were each sub-cultured twice in de Man Rogosa and Sharpe (MRS) broth (Merck, Darmstadt, Hesse, Germany), incubated at 37 °C for 72 h in anaerobic jars containing Anaerocult A gaspacks with Anaerotest strips (Merck, Darmstadt, Hesse, Germany). The cultures were standardized to an optical density of 0.2 at 600 nm for use in experiments.

### 2.2. Categorization of L. monocytogenes Strains as Biofilm Formers

Overnight cultures of each *L. monocytogenes* strain (Avo, Cuc, 243 and ATCC 19115) were prepared by inoculating 200 µL of each cultures into 10 mL of BHI, and then incubated at 37 °C for 18 h. The optical density of the cultures was adjusted to 0.2–0.25 at 594 nm. Then 200 µL of each culture was transferred to separate wells of the 24-well clear polyvinyl chloride (PVC) microtiter plates in triplicate. The BHI medium was added to additional three wells to serve as the negative control. The plates were incubated for 48 h aerobically at 37 °C for biofilms to form. The biofilms formed in the wells were quantified according to methods of Djordjevic et al. [24] and Gómez et al. [25], with minor modifications. Briefly, the medium in the wells was discarded and then loosely attached cells were washed from the wells using 2 mL of ¼ strength Ringer’s solution. The cells attached to the wells were gently washed thrice with sterile distilled water and thereafter the plates were emptied, inverted and allowed to dry for 30 min. Each well was treated with 150 μL of 1% crystal violet dye and left to stand for 45 min at room temperature. Excess dye was washed off five times with sterilized water and then the wells were solubilized and destained with 200 μL of 95% ethanol, at 4 °C for 30 min. After the 30 min, 200 μL of the well contents were transferred to a new sterile PVC microtiter plate. The absorbance of the wells was measured using a SpectraMax^®^ Paradigm^®^ Multi-Mode Detection Platform microtiter plate reader at 594 nm (OD_595_). The *L. monocytogenes* strains were then classified as either a non-biofilm, weak, moderate or a strong biofilm producer according to [26] as follows: non-biofilm producers (OD ≤ ODC), weak biofilm producer (ODC < OD ≤ 2 × ODC), moderate biofilm producer (2 × ODC < OD ≤ 4 × ODC) or strong biofilm producer (4 × ODC < OD). The ODC was 0.05.

### 2.3. Preparation of Cell Free Supernatants (CFS) of LAB

For each lactobacilli, 200 µL was inoculated into 10 mL of MRS broth in a glass test tube and incubated at 37 °C for 24 h in anaerobic jars containing Anaerocult A gaspacks with Anaerotest strips. Then the cell free supernatants (CFS) were prepared using the method of [27], without modifications. Briefly, the culture was centrifuged at 4000× *g* for 10 min at 20 °C and the supernatant was filtered through a cellulose nitrate filter of 0.2 µm. The CFS were used in experiments in undiluted form.

### 2.4. Biofilm Formation in Microwell Plates by L. monocytogenes in the Presence of CFS of LAB

An overnight culture of each *L. monocytogenes* strain was prepared by inoculating 200 µL of the strain into 10 mL of BHI broth separately and incubated aerobically for 18 h at 37 °C. From the overnight culture, 200 µL was transferred to each of the twelve wells of a 24 well microtiter plate. Subsequently, 2 mL of each CFS was added to the wells containing the *L. monocytogenes* cultures in triplicate. The three wells to which no CFS was added served as the positive control while additional three wells containing 200 µL BHI served as the negative control. The microtiter plate was incubated aerobically at 37 °C for 48 h, washed to remove excess media and unbound cells, and then the biofilms were quantified according to the method by Djordjevic et al. [24] and Gómez et al. [25]. Each experiment was repeated in three independent trials, with each treatment done in triplicate.

### 2.5. Dispersion of Preformed L. monocytogenes Biofilms in Microwell Plates by CFS of LAB

Individual *L. monocytogenes* strains were allowed to form biofilms in microtiter well plates as described under Section 2.2. Then 2 mL of CFS of each LAB were separately added to the wells with preformed biofilm in triplicate, then the plates were incubated at 37 °C for a further 48 h. The microtiter plates were washed and biofilms quantified according to the methods by Djordjevic et al. [24] and Gómez et al. [25].

### 2.6. Dispersion of Preformed L. monocytogenes Biofilms on Stainless Steel and PVC Coupons by CFS of LAB

#### 2.6.1. Preparation of *L. monocytogenes* Bacterial Suspensions

Preparation of the *L. monocytogenes* cultures and inoculation of coupons was done according to the method of Milanov et al. [28], with modifications. Briefly, overnight BHI broth cultures of *L. monocytogenes* strains (ATCC 19115 and 243) were prepared, and serially diluted up to 10^−6^ dilution using ¼ strength Ringer’s solution. Then 100 µL of the 10^−4^, 10^−5^ and 10^−6^ dilutions were spread plated onto BHI agar plates, and the plates were incubated at 37 °C for 24 h. Subsequently, three colonies from the BHI agar plates were inoculated into 3 mL of Tryptic soy broth supplemented with 0.6% yeast extract (TSB-YE) in a glass test tube. The test tubes were incubated for 24 h at 25 °C. The optical density of the inoculum was adjusted to 0.2–0.25 at 594 nm before use in experiments.

#### 2.6.2. Biofilm Formation on Stainless Steel and PVC Coupons

The stainless steel was cut out into 2 cm × 2.5 cm rectangular coupons while PVC was cut out into circular coupons with a radius of 2 cm. The coupons were boiled in water for 5 min, then soaked for a further 5 min in 5% sodium hypochlorite solution at room temperature and subsequently rinsed five times with distilled water. They were then immersed in 100% ethanol and passed through a flame prior to their use in the experiments. The coupons were placed into separate wells (1 coupon per well) of sterile polystyrene 6-well plates, to which 100 µL of *L. monocytogenes* ATCC 19115 and 243 bacterial suspensions were individually transferred, and then the microwell plates were incubated at 25 °C for 3 h. Then non-adherent bacteria were removed from the wells by pipetting and washing with 3 mL of sterile Phosphate-buffered saline (PBS). Subsequently, 300 µL of CFS of each LAB was added and the plates was incubated for 1 h at 25 °C. Then 5 mL of sterile TSB-YE were added to each well and the plates incubated for 7 days at 25 °C. On every second day the old broth from the wells was replaced with 5 mL of fresh TSB-YE.

#### 2.6.3. Scanning Electron Microscopy

Following 7 days of incubation the stainless steel and PVC coupons were removed from the wells and washed by mild pipetting with 3 mL of sterile PBS to remove the medium and non-adherent cells. The coupons were then prepared for microscopy according to the method of Booyens et al. [29], with minor modifications. Briefly, the coupons were fixed using 2.5% glutaraldehyde in 0.075 mol^−1^ phosphate buffer (pH 7.4) for 30 min. They were subsequently washed three times in 0.15 mol^−1^ PBS before being dehydrated in a series of graded alcohol concentrations (30%, 50%, 70%, 90% and 100% ethanol) for 15 min each, and then in 100% ethanol for 30 min. They were then submerged in a 50:50 hexamethyldislazane (HMDS) and 100% ethanol solution for 1 h. The solution was removed and coupons were treated with HMDS for 1 h. The old HDMS was replaced with a fresh one and the coupons were left to air dry. The cells were critically dried for 24 h before being coated with carbon. The stainless steel coupons were directly coated with carbon while the PVC coupons were first mounted onto aluminum stubs and then carbon coated. They were then viewed using a Zeiss Crossbeam 540 FEG and Zeiss 540 Ultra scanning electron microscope.

### 2.7. Quantification of prfA Gene Expression by L. monocytogenes

*L. monocytogenes* 243 was sub-cultured into BHI broth in test tubes at 37 °C for 18 h, the culture density was adjusted to OD_600_ = 0.2. Then 2 mL of the CFS of each LAB were separately added to the cultures. The *L. monocytogenes* culture to without CFS served as the control. Total RNA was extracted from the cultures after 24 h at 37 °C using the PureLink ^®^ RNA Mini Kit with Trizol^®^ reagent (Thermo Scientific, Waltham, MA, USA) according to the manufacturer’s instructions. The RNA was eluted with RNase-Free water and quantified using the NanoDrop™ 2000 spectrophotometer (Thermo Scientific, Waltham, MA, USA) and then stored at −80 °C. Subsequently, cDNA was synthesized from 2 µL total RNA of control and treated samples using the Maxima H Minus First Strand cDNA Synthesis Kit (Thermo Scientific, Waltham, MA, USA) following the manufacturer’s instructions, which was optimized to generate first strand cDNA for use in two-step RT qPCR. The quality was assessed using the NanoDrop™ 2000 spectrophotometer.

The primers (Table 1) were designed using Basic Local Alignment Search Tool (BLAST) in combination with the Primer Design 4.1. Quantitative reverse transcriptase polymerase chain reaction (RT qPCR) was conducted using PowerUp™ SYBR™ Green Master Mix (Applied Biosystems, Foster City, CA, USA) according to the protocol’s reaction set-up. Reactions were carried out in QuantStudio™ 5 Real-Time PCR System 384-well block (Applied Biosystems, Foster City, CA, USA). The RT-qPCR reaction mix was set at 10 μL with 5 μL of SYBR Green reference dye, 1.5 μL nuclease-free water, 0.5 μL of each primer and 2.5 μL of cDNA template. The standard cycling parameters consisted of 50 °C for 2 min and 95 °C for 2 min holding cycles for UDG activation and Dual-Lock™ DNA polymerase, respectively. This was followed by 40 cycles of 95 °C for 15 sec of denaturation and 56 °C for 1 min anneal/extend stage, with the fluorescent signal collected at the extension step. The experiment was performed in biological triplicates and technical quadruplicates. Relative gene expression was determined using the Pfaffl [30] method, with a slight modification of incorporating the geometric average of all relative quantities of the multiple reference genes used.

### 2.8. Statistical Analysis

All the experiments were performed in triplicates in three independent trials. The values reported are averages and standard error of the means. The software GraphPad Prism 8.4.1 was used to analyze the results to perform the two-way ANOVA (Analysis of Variance) followed by the Tukey’s multiple comparisons test (*p* < 0.05).

## 3. Results and Discussion

### 3.1. Biofilm Formation Profiles of the Test L. monocytogenes Strains

**Figure 1:**
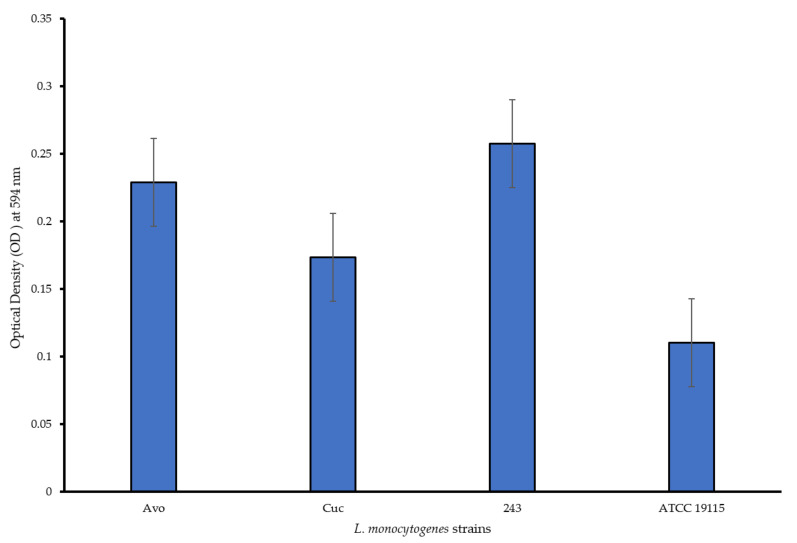
Biofilm formation capabilities of the test *L*. *monocytogenes* strains in PVC microtiter plates.

All the *L. monocytogenes* strains formed biofilms within the microtiter wells, with the different strains displaying varied strengths of biofilm production (Figure 1). Based on the Borges et al. [26] biofilm classification system, *L. monocytogenes* Avo and *L. monocytogenes* 243 strains were classified as strong biofilm producers while *L. monocytogenes* Cuc and *L. monocytogenes* ATCC 19115 were classified as moderate biofilm producers. Overall all the *L. monocytogenes* strains isolated from the food environments were stronger biofilm formers than the *L. monocytogenes* ATCC 19115 strain. Researchers elsewhere reported the differences in biofilm-forming capacities of *L. monocytogenes* strains [7,9]. The differences exhibited with regards to biofilm formation abilities of *L. monocytogenes* strains is dependent on multiple factors including the serotype of the strain, which identifies based on cells surface antigens [31]. The phenotype of the biofilm is reportedly related to the clonal lineage due to specificities in the qualitative, quantitative and dynamic features expressed by the specific strain [32]. The ability of the *L. monocytogenes* isolates from avocados and cucumbers to form biofilms is a cause for concern as it indicates their potential to persist in their respective environments and consequently pose a food safety risk due to their contamination of these produce. *L. monocytogenes* has been reported to form biofilms on apples, lettuce and cucumber [18,19,20,21].

### 3.2. Biofilm Formation Capabilities of L. monocytogenes Strains in the Presence of CFS of LAB

**Figure 2:**
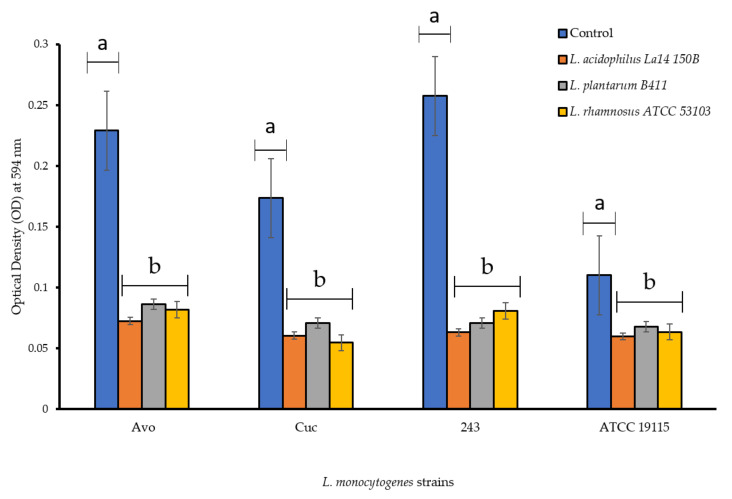
Biofilm formation capabilities of L. monocytogenes strains in PVC microtiter plates in the presence of individual cell free supernatants of lactic acid bacteria. Each bar represents the mean of triplicates from three separate trials and the error bars show the standard error. Bars represented with different letters are statistically different (*p* < 0.05), while those with the same letter have no statistical differences (*p* > 0.05).

Biofilm formation capabilities of *L. monocytogenes* strains when grown in the presence of CFS of LAB are shown in Figure 2. The strains from avocados and cucumber were superior biofilm formers than the ATCC strain. The presence of CFS of LAB negatively affected biofilm formation capabilities of all *L. monocytogenes* strains, which was evident because in the absence of these treatments the strains formed dense/mature biofilms. All the *L. monocytogenes* strains were downgraded post CFS treatment and were classified into weaker (lesser biofilm formation categories compared to in the absence of CFS treatment) biofilm producer categories (Figure 2), indicating anti-biofilm abilities of the CFS. *L. monocytogenes* Avo and 243 which were originally classified as strong biofilm formers in absence of CFS were subsequently categorized as weak biofilm formers, while *L. monocytogenes* Cuc and *L. monocytogenes* ATCC 19115 were demoted from their moderate biofilm former status to a weaker category in the presence of all CFS treatments. Notably, *L. rhamnosus* ATCC 53103 CFS (pH 4.2) decreased the optical density measured at wavelength 594 nm (OD_595_) of *L. monocytogenes* Cuc to below 0.05 meaning no biofilm formation occurred at all. *L. monocytogenes* ATCC 19115 was decreased from a moderate to a weak biofilm producer across all treatments too. Overall, *L. acidophilus* La14 150B (pH 3.8) was the most effective with regards to inhibition of biofilm formation across all *L. monocytogenes* strains. This was concluded by the lower OD_595_ values recorded after treatment with *L. acidophilus* La14 150B and showed prominent inhibitory effects. *L. plantarum* B411 CFS (pH 4.2) was the least effective of all the LAB in inhibiting biofilm formation, however, it still managed to change the classification of all the *L. monocytogenes* strains into a weaker category compared to the control (Figure 2). There were significant statistical differences (*p* < 0.05) between the OD_595_ values post-treatment with all three CFS in comparison to the control *L. monocytogenes* strains. Compared to the control, all CFS significantly inhibited biofilm formation by *L. monocytogenes* strains (*p* < 0.05). However, there were no significant differences in inhibition of the biofilm formation by the CFS of the different LAB (*p* > 0.05).

### 3.3. Dispersion of Preformed L. monocytogenes Biofilms by CFS of LAB

**Figure 3:**
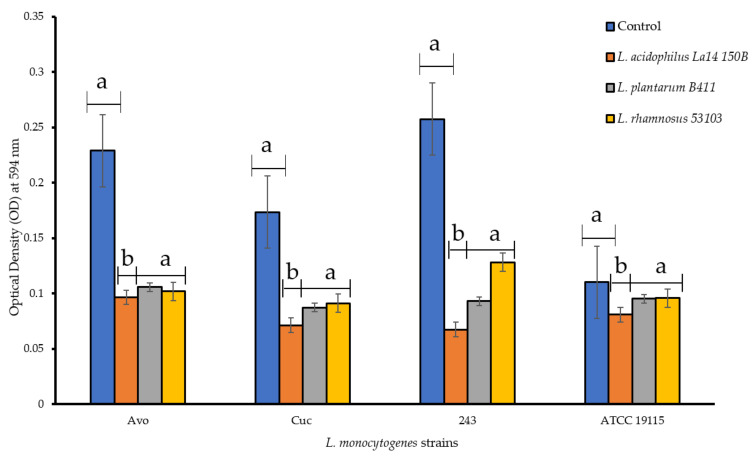
Dispersion of preformed *L. monocytogenes* biofilms in PVC microtiter plates by cell free supernatants of individual lactic acid bacteria. Each bar represents the mean of triplicates from three separate trials and the error bars represent standard error. Bars represented with different letters are statistically different (*p* < 0.05), while those with the same letter have no statistical differences (*p* > 0.05).

The ability of CFS to disperse *L. monocytogenes* biofilms already formed within the microtiter plates was investigated. Following the treatments with the CFS, the preformed *L. monocytogenes* biofilms were not completely dispersed but were classified into weaker biofilm forming categories. Individual CFS of *L. plantarum* B411 and that of *L. rhamnosus* ATCC 53103 reduced the biofilm forming category of *L. monocytogenes* Avo from strong to moderate, while CFS of *L. acidophilus* La14 150B reduced it to a weak biofilm former (Figure 3). Both *L. monocytogenes* Cuc and ATCC 19115 strains were demoted from the moderate biofilm former category to a weak biofilm former after treatment with individual CFS of all LAB. Only the CFS of *L. acidophilus* La14 150B significantly dispersed biofilms formed by all the *L. monocytogenes* strains (*p* < 0.05). The efficiency of this CFS in disruption of biofilms was also significantly higher than those of the other LAB, while there we no significant differences between efficiencies of CFS of *L. plantarum* B411 and *L. rhamnosus* ATCC 53103 (*p* > 0.05). Overall, CFS of *L. acidophilus* La14 150B was the most efficient while that of *L. rhamnosus* ATCC 53103 was the least efficient. None of the CFS of the tested LAB was able to completely disperse pre-formed biofilms of any of the *L. monocytogenes* strains.

### 3.4. The Effect of CFS of LAB on L. monocytogenes Biofilms Preformed on Stainless Steel and PVC Coupons

**Figure 4:**
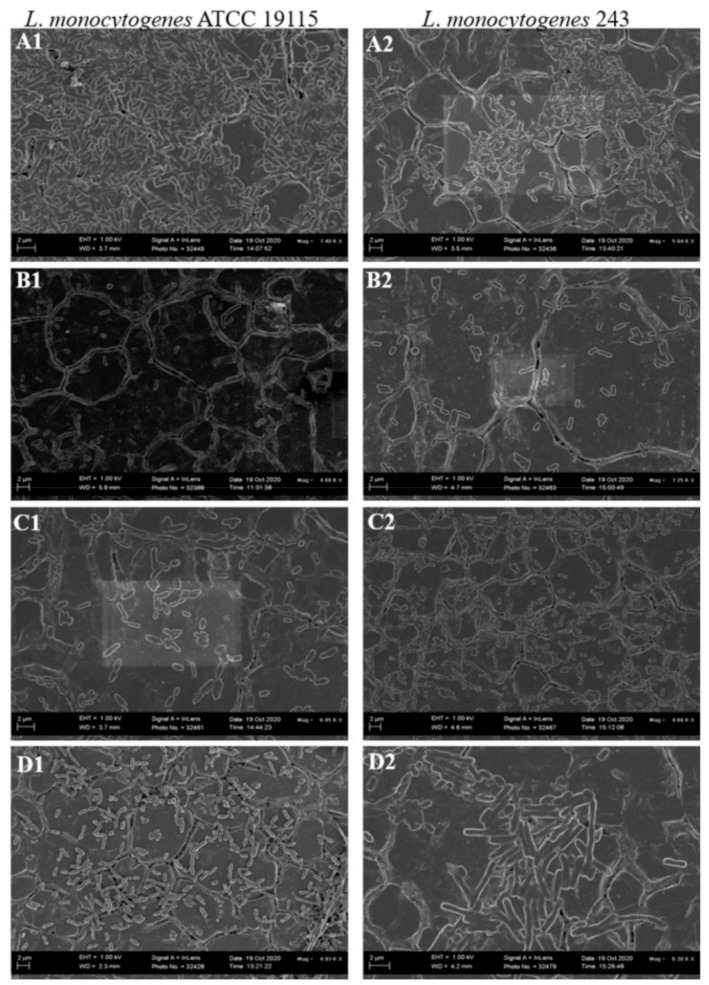
Scanning electron microscopy images of *L. monocytogenes* ATCC 19115 and *L. monocytogenes* 243 biofilms on stainless steel coupons after 7 days of incubation in TSB at 25 °C (**A**), control; and after 1 h treatment with CFS of (**B**), *L. acidophilus* La14 150B; (**C**), *L. plantarum* B411; (**D**), *L. rhamnosus* ATCC 53103.

*L. monocytogenes* forms biofilms on a variety of surfaces used in the food industry, such as polytetrafluoroethane, polyster, polystyrene, rubber, stainless steel used in conveyor belts, floor sealers, drain materials, joints, and equipment, respectively, as well as on wood and glass [11,33]. In light of these, this study investigated the effect of CFS of test LAB on biofilms of *L. monocytogenes* pre-formed on stainless steel and PVC coupons. Both *L. monocytogenes* ATCC 19115 and *L. monocytogenes* 243 formed mature biofilms on stainless steel (Figure 4A). The architecture of the biofilms on stainless steel coupons resembled that of biofilms formed by majority of listerial strains, forming honeycomb-like structures with layers of cohesive cells [10]. Treatment of the coupons with CFS of LAB resulted in disruption of the structural integrity of the biofilm, evidenced by disentanglement of the cells and an increase in the number of isolated cells on the coupon surfaces (Figure 4B,D). The anti-biofilm efficacy of the different CFS varied, with that of *L. acidophilus* La14 150B being the most potent while that of *L. rhamnosus* ATCC 53103 was the weakest as the biofilms of both *L. monocytogenes* strains remained somewhat intact even after exposure to this CFS. This result correlated with the results obtained for biofilms formed in microtiter well plates.

**Figure 5:**
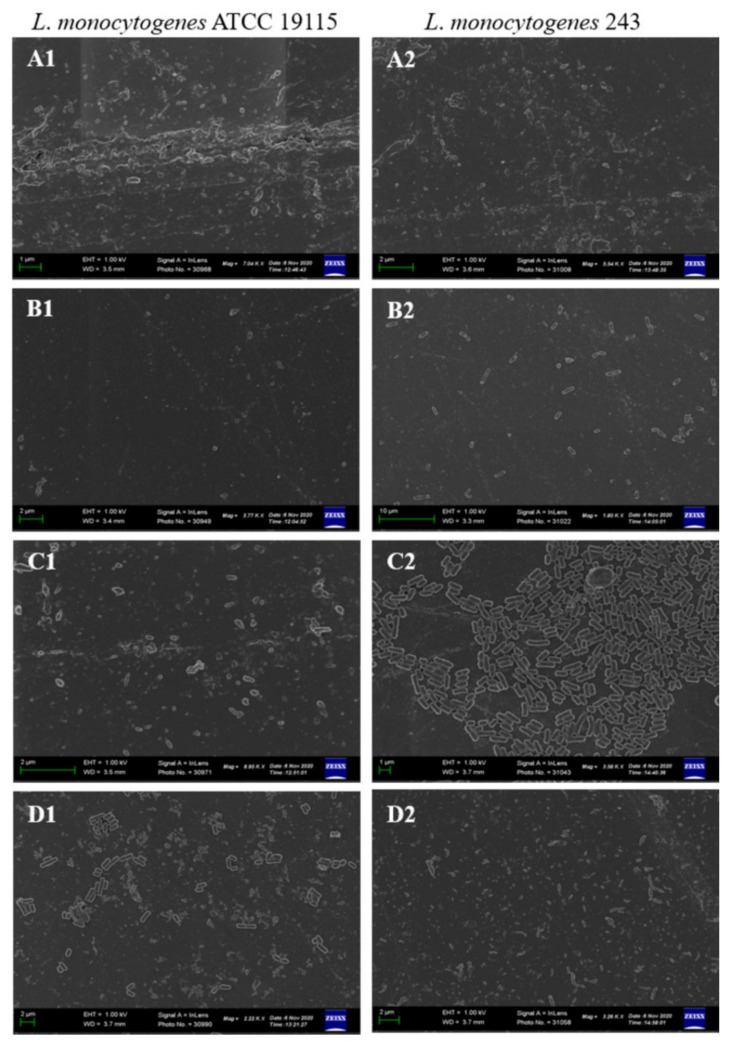
Scanning electron microscopy images of *L. monocytogenes* ATCC 19115 and *L. monocytogenes* 243 biofilms on PVC coupons after 7 days of incubation in TSB at 25 °C (**A**), control; and after 1 h treatment with CFS of (**B**), *L. acidophilus* La14 150B; (**C**), *L. plantarum* B411; (**D**), *L. rhamnosus* ATCC 53103.

Similar to what was observed on stainless steel coupons, both *L. monocytogenes* strains formed biofilms on PVC coupons. However, the biofilm visual architecture differed to that formed on stainless steel coupons, with the biofilm appearing as a dense mass of a monolayer of adherent cells, without visible honey-comb structures. (Figure 5A). These differences could be attributed to the properties of the different coupons as the type and characteristics of the surface are among others, crucial factors affecting biofilm formation [28]. Treatment with CFS interfered with and disrupted aggregation of cells in the biofilm, resulting in appearance of scattered rod shaped cells (Figure 5C,D). In consistency with what was observed for biofilms preformed onto the stainless steel coupons, CFS of *L. acidophilus* La14 150B exhibited the highest anti-biofilm activity while CFS of *L. rhamnosus* ATCC 53103 was the least effective.

LAB can directly attack physical membrane, disfigure the biofilm structure and interrupt the protein confirmations of the pathogen [34]. They engage in a diverse range of active competitive strategies to achieve dispersal including, among others, production of antimicrobial compounds and metabolites, interfering with the competitors signaling and motility; and by directly forcing the dispersal of the competitor using biosurfactants produced [35,36]. The anti-biofilm effects of the CFS of test LAB could be attributed to the presence of these compounds as they are released into the medium. In a recent related study, the anti-biofilm effect of CFS against *L. monocytogenes* biofilms was attributed to presence of surfactants in the CFS of probiotic *Saccharomyces cerevisiae* [4]. Biosurfactants directly interfere with membrane functions and energy generating structures, decreasing the cell surface hydrophobicity, which reduces the ability of microbes to adhere to the surface [34]. The enhanced anti-biofilm potency of *L. acidophilus* La14 150B could be due to its production of biosurfactants, which accelerates dispersal of biofilms and modify their structural parameters [37]. Jara et al. [38] reported that *Lactobacillus* interfered with the synthesis of EPS and distribution of species within the biofilms of *L. monocytogenes*. This is another possible mechanism by which CFS of LAB disrupted the *L. monocytogenes* biofilms, however this mechanism of natural immobilization for CFS needs to be further investigated.

### 3.5. prfA Gene Expression

**Figure 6:**
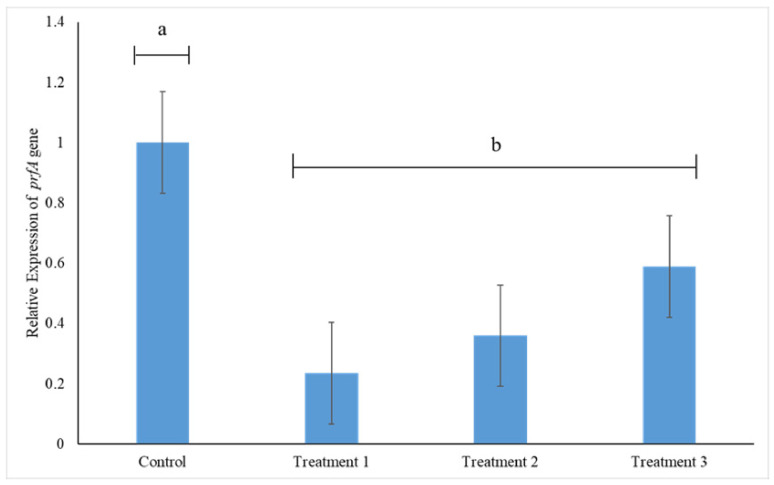
Relative *prfA* gene expression in *L. monocytogenes* 243 treated with cell free supernatants (CFS) of *L. acidophilus* La14 150B (Treatment 1), *L. plantarum* B411 (Treatment 2) and *L. rhamnosus* ATCC 53103 (Treatment 3). Bar heights indicate mean expression of the gene in triplicate samples while error bars indicate standard error. Bars with different letters are significantly different (*p* < 0.05).

The expression of *prfA* gene, which codes for the major regulon PrfA, was significantly reduced in the presence of CFS of all LAB strains (*p* < 0.05) (Figure 6). Taking the expression of *prfA* gene in the control to be 100%, its expression was downregulated by 77%, 64% and 41%, due to the presence of CFS of *L. acidophilus* La14 150B (Treatment 1), *L. plantarum* B411 (Treatment 2) and *L. rhamnosus* ATCC 53103 (Treatment 3), respectively. Thus CFS of *L. acidophilus* La14 150B induced the most negative effect on *prfA* gene expression, although not significantly different to the effects by the CFS of the other two LAB strains (*p* > 0.05) (Figure 6).

The disruptions caused by CFS can be attributed to a wide range of properties including among others, the presence of antimicrobial compounds, mostly organic acids (lactic and acetic acids) and bacteriocins [39]. Organic acids target specific metabolic functions including replication and aggregation of cells, leading to premature death [40,41]. They also generate a selective barrier that alters cell metabolism and virulence progression, damage enzymes and the genetic material [42]. The pronounced downregulation of *prfA* gene expression by *L. acidophilus* La14 150B could be due to the fact that *L. acidophilus*, as previously shown by Liguori et al. [43], yields the highest level of lactic acid among other *Lactobacillus* strains. However, considering that the pH values of the CFS of the test LAB were slightly different although their anti-biofilm potencies were different, especially the CFS of *L*. *acidophilus* La14 150B versus those of the other two LAB, the low pH cannot be the sole mechanism of action employed. This is further underscored by varied efficiencies between the CFS of *L*. *plantarum* B411 and *L*. *rhamnosus* 53103, which had the same pH. PrfA of *L. monocytogenes* has a significant impact on extracellular biofilm formation, with mutants lacking it being defective in surface-adhered biofilm formation [6]. Thus, normal expression of *prfA* promotes the aggregation and formation of biofilms. In concurrence with majority of published literature, our results suggest that one of the possible mechanisms by which CFS of the tested LAB inhibit and/or disperse *L. monocytogenes* 243 biofilms is through downregulation of *prfA* gene expression. The downregulation of *prfA* can play a role towards reducing the virulence of *L*. *monocytogenes* as it is involved in regulation of other pathogenesis-related genes. However, this has to be treated with caution as a recent study by Bai et al. [7] reported a 25% downregulation of *prfA* gene expression by *L. monocytogenes* biofilm cells in the absence of any antimicrobial treatment. The implications of their study could then be that the observed downregulation of *prfA* gene could be due to their sessile form, but not necessarily a direct link between biofilm formation capability of *L. monocytogenes* and *prfA* gene expression.

## 4. Conclusions

*Listeria monocytogenes* strains isolated from avocado and cucumber fruits and processing environments have the capability to form biofilms on different simulated food contact surfaces. Cell free supernatants (CFS) of selected lactic acid bacteria possess anti-biofilm activities with varied potencies against these biofilms. One of the potential mechanisms of anti-listerial biofilm inhibition and dispersion by these CFS is through downregulation of *prfA* gene, known to be involved in biofilm formation by *L*. *monocytogenes*. The results of this study are of importance to the avocado and cucumber food processing facilities and the food industry at large as it provides evidence for the potential of CFS of probiotic LAB as a safe alternative anti-biofilm agent that can be used to control *L*. *monocytogenes* biofilms on these fresh produce. Application of these CFS as part of the antimicrobial regimes will minimize contamination of the avocados and cucumbers by *L*. *monocytogenes*, consequently lessening their chances of acting as vehicles for transmission of this pathogen to consumers.

## Figures and Tables

**Table 1:** List of primer sequences used in this study.

Gene	GenBank^®^ Accession Number	Primers Sequences (5′ to 3′)		Length (bp)
		Forward	Reverse	
*prfA* (Gene of interest)	JN703898.1	tagcgagaacgggaccatca	aacgtatgcggtagcctgct	136
*GAPDH* (Reference gene)	FJ890134.1	aggtgacttccgtcgtgcac	gaacacgttgagcagctccg	128
*bgla* (Reference gene)	FM180366.1	cggtcacattactgacggtcc	ggaagatacgggaccaagcga	146

*pfrA*: Positive regulatory factor A; *GAPDH*: Glyceraldehyde-3-phosphate dehydrogenase; *bgla*: beta-glucosidase.

## Data Availability

Data are contained within the article.
